# Reconstruction-Dependent Recovery from Anorexia and Time-Related Recovery of Regulatory Ghrelin System in Gastrectomized Rats

**DOI:** 10.1155/2010/365416

**Published:** 2010-02-24

**Authors:** Masaru Koizumi, Katsuya Dezaki, Hiroshi Hosoda, Boldbaatar Damdindorj, Hideyuki Sone, Lu Ming, Yoshinori Hosoya, Naohiro Sata, Eiji Kobayashi, Kenji Kangawa, Hideo Nagai, Yoshikazu Yasuda, Toshihiko Yada

**Affiliations:** ^1^Division of Integrative Physiology, Department of Physiology, Jichi Medical University School of Medicine, Yakushiji 3311-1, Shimotsuke, Tochigi 329-0498, Japan; ^2^Department of Surgery, Jichi Medical University School of Medicine, Yakushiji 3311-1, Shimotsuke, Tochigi 329-0498, Japan; ^3^Department of Biochemistry, National Cardiovascular Center Research Institute, 5-7-1 Fujishirodai, Suita, Osaka 565-8565, Japan; ^4^Division of Organ Replacement Research, Center for Molecular Medicine, Jichi Medical University, Yakushiji 3311-1, Shimotsuke, Tochigi 329-0498, Japan; ^5^Department of Surgery, Ibaraki Prefectural Central Hospital, Koibuchi 6528, Kasama, Ibaraki 309-1793, Japan

## Abstract

Gastrectomy reduces food intake and body weight (BW) hampering recovery of physical conditions. It also reduces plasma levels of stomach-derived orexigenic ghrelin. This study explored changes in orexigenic ghrelin system in rats receiving total gastrectomy with Billroth II (B-II) or Roux-en-Y (R-Y) method. Feeding and BW were reduced by gastrectomy and subsequently recovered to a greater extent with R-Y than B-II while plasma ghrelin decreased similarly. At postoperative 12th week, ghrelin contents increased in the duodenum and pancreas, plasma ghrelin levels increased upon fasting, and ghrelin injection promoted feeding but not in earlier periods. In summary, gastrectomized rats partially recover feeding and BW, in a reconstruction-dependent manner. At 12th week, ghrelin is upregulated in extra-stomach tissues, plasma ghrelin levels are physiologically regulated, and orexigenic effect of exogenous ghrelin is restored. This time-related recovery of ghrelin system may provide a strategy for promoting feeding, BW, and thereby physical conditions in gastrectomized patients.

## 1. Introduction

Ghrelin, a 28-amino acid peptide, is produced mainly in the stomach and to a lesser extent in the small intestine, pancreas, and hypothalamus and is the endogenous ligand for the growth hormone secretagogue receptor (GHS-R) [1–9]. Serine-3 of ghrelin is acylated with an octanoic acid, the process catalyzed by a recently discovered ghrelin *O*-acyltransferase (GOAT) [10, 11]. The acylation is thought to be required for its biological activity [1], although desacyl ghrelin has been reported to exert several effects [12]. Administration of pharmacological doses of acylated ghrelin (= ghrelin) to intact animals increases food intake, induces body weight gain, and causes obesity [13–18]. The orexigenic and body fat promoting properties of ghrelin and growth hormone (GH) secretagogue (GHS) are thought to be independent of GH and mediated primarily by the hypothalamic neuropeptide Y (NPY) and agouti-related protein (AGRP) systems [14–16, 19–22]. Although pharmacologic effects of ghrelin are well documented, physiological role of endogenous ghrelin is poorly understood. It has recently been reported that knockout of either the ghrelin gene or ghrelin receptor gene exerts no or minor effects on body weight and body composition [23–25]. However, the lack of phenotypic changes in knockout mice might reflect compensatory mechanisms that are known to operate occasionally.

In humans, gastrectomy results in loss of body weight of about 10% within the first six months after surgery, primarily due to reduced body fat [26]. In addition, gastrectomized patients often complain of loss of appetite, general fatigue, and in some cases impaired bone quality such as osteopenia and osteomalacia [9, 27]. At present there are no satisfactory mechanistic explanation and treatment for any of these symptoms. The effects of gastrectomy on food intake and body composition have been poorly documented in rodents. Loss of ghrelin could be implicated in these symptoms, since as much as 80% of circulating ghrelin is lost following surgical removal of the glandular stomach or the acid producing part of the stomach in rats and humans [4, 28, 29].

In this study, we performed total gastrectomy in rats and examined whether the recovery from gastrectomy-associated anorexia depends on the circulating ghrelin level or other factors and studied temporal changes in the biosynthesis and orexigenic ability of ghrelin following gastrectomy. 

## 2. Materials and Methods

### 2.1. Animals and Gastrectomy

Male 4-week-old Wistar rats (SLC, Japan) were maintained on a 12-hour light/dark cycle and given conventional food and water for 2 weeks and not deprived of food before gastrectomy. They were operated at 6 weeks of age with body weight around 130–180 g. Rats were anesthetized with an intraperitoneal injection of pentobarbital (40 mg/kg) and a median abdominal incision was made. After the stomach was separated from the greater and lesser omentum, the duodenal bulb was ligated and transected. After the left gastric artery was ligated, the esophagus was clamped above the esophagogastric junction and the stomach was resected. An end-to-side anastomosis between esophagus and jejunum at the 4-5 cm anal side from Treitz ligament was performed with 7–0 monofilament polyglyconate synthetic absorbable string (Maxon; Johnson & Johnson Inc. USA) using interrupted suture (Billroth II (B-II) reconstruction). On the other hand, jejunum was transected at the 2-3 cm anal side from the Treitz ligament. Then, an end-to-side anastomosis between the esophagus and jejunum was performed with 7–0 Maxon using an interrupted suture. Jejunojejuno anastomosis was also done by end-to-side method (Roux-en-Y (R-Y) reconstruction). The abdominal wall and skin closure was made by 5–0 Nylon (Johnson & Johnson Inc. USA) in a running suture. After the operation, the rats were allowed only clear water without food for 3 days, dry milk from the 3rd day, and from the 7th day a conventional pellet diet with free access to water. The care of the animals was in accordance with our institutional guidelines.

### 2.2. Measurements of Food Intake and Body Weights

After the operation, food intake for 24 hours and body weight were measured once a week for 3 months.

### 2.3. Preparation of Blood and Tissue Samples

The rats were sacrificed at three months after operation. To measure plasma ghrelin concentrations, blood samples were collected from the inferior vena cava of anaesthetized rats. Duodenum samples of about 3 cm were taken from 1 cm anal side of duodenal stump. Jejunum samples of about 3 cm were taken from 1 cm anal side of esophagojejunostomy. Pancreatic samples were also taken. The tissues were quickly frozen and stored at −80°C until assaing.

### 2.4. RIAs Using Specific Antiserum and ELISA for Ghrelin

 Two kinds of rabbit polyclonal antiserum were used in the present study. One antiserum was raised against the COOH-terminally Cys-extended rat ghrelin (position 1-11) in New Zealand white rabbits (#G606) that was shown to specifically recognize ghrelin with *n*-octanoylated Ser 3 (acylated ghrelin; ghrelin). By the radioimmunoassay (RIA) using this antiserum, designated NH_2_-terminal RIA (N-RIA), the concentration of acylated ghrelin was obtained. The other antiserum was raised against the NH_2_-terminally Cys-extended rat ghrelin (position 13-28) (#G107) that was shown to recognize both acylated ghrelin and desacyl ghrelin [3]. By the RIA using this antiserum, named as COOH-terminal RIA (C-RIA), the concentration of acylated ghrelin plus desacyl ghrelin was obtained.

These two ghrelin-specific RIAs were used to measure acylated ghrelin and desacyl ghrelin contents in several tissues [3]. The bound and free ligands were separated using a second antibody. “Acylated ghrelin” is expressed as “ghrelin” and “acylated ghrelin plus desacyl ghrelin” is expressed as “total ghrelin” throughout this text.

Plasma concentrations of acylated ghrelin and desacyl ghrelin were measured using ELISA kits (Mitsubishi Kagaku Iatron, Tokyo, Japan).

### 2.5. Treatment with Ghrelin

Ghrelin (Peptide Institute, Osaka, Japan) or saline was administered subcutaneously to control normal rats (eight-weeks-old) and those received gastrectomy with B-II reconstruction. For ghrelin treatment at 2 and 6 weeks after gastrectomy, the following procedures were used. Control and gastrectomized rats were divided into two groups; one group was treated with ghrelin and the other with saline, and food intake and body weight were measured. The injection of ghrelin (10 nmol/kg body weight) was carried out once a day for seven days, and food intake and body weight were measured. For ghrelin treatment at 12 weeks after gastrectomy, the following procedures were used. Single injection of ghrelin or saline was followed by measurements of food intake for the subsequent 24 hours. In the next week, two groups were changed: one group that had first received ghrelin was injected with saline, and the other group that had first received saline was injected with ghrelin. Food intake after the first injection and that after the second injection were pooled and averaged.

### 2.6. Statistical Analysis

 Data represent the means ± SEM (*n* = number of rats). Statistical analysis was performed using Student's paired and unpaired *t*-tests. Values of *P* < .05 were considered statistically significant.

## 3. Results

### 3.1. Food Intake and Body Weight in Total Gastrectomized Rats

**Figure 1:**
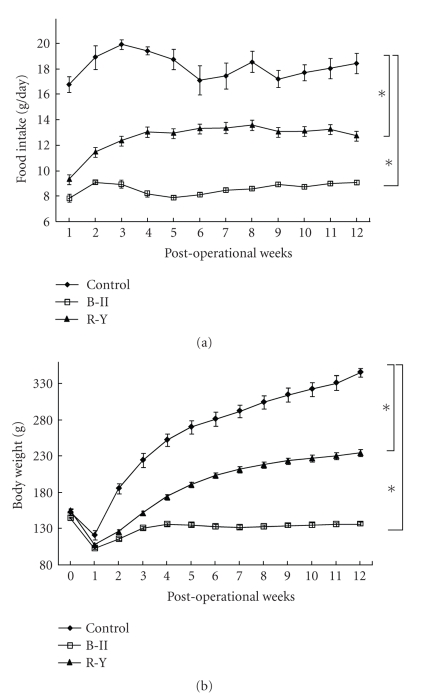
Daily food intake (a) and body weight (b) in rats for 12 weeks after total gastrectomy combined with Billroth II (B-II) or Roux-en-Y reconstruction method (R-Y), and after sham-operation as the control. Gastrectomy was performed at 6 weeks of age. *N* = 6 for control, 40 for B-II and 35 for R-Y. *P<.05.

Food intake for 24 hours and body weight were measured in total gastrectomized and control rats during 12 postoperative weeks. Gastrectomy with B-II (*n* = 40) and R-Y reconstruction methods (*n* = 35) both markedly decreased food intake. In both B-II and R-Y groups, at the 1st week after operation food eaten for 24 hours decreased to a level around 50% of that in control rats (Figure 1(a)). In B-II group the reduced level of food intake continued for 12 weeks after operation. In contrast, food intake in R-Y group increased gradually during 4 postoperative weeks. The daily food intake averaged for postoperative 12 weeks was significantly (*P* < .05) reduced in B-II and R-Y groups (Figure 1(a)). Food intake in R-Y group was significantly (*P* < .05) greater than in B-II group throughout postoperative 12 weeks. Body weight significantly decreased after gastrectomy in both B-II and R-Y groups (Figure 1(b)). Body weight in R-Y group was significantly greater than that in B-II group from the 2nd through 12th postoperative week (*P* < .05). 

**Figure 2:**
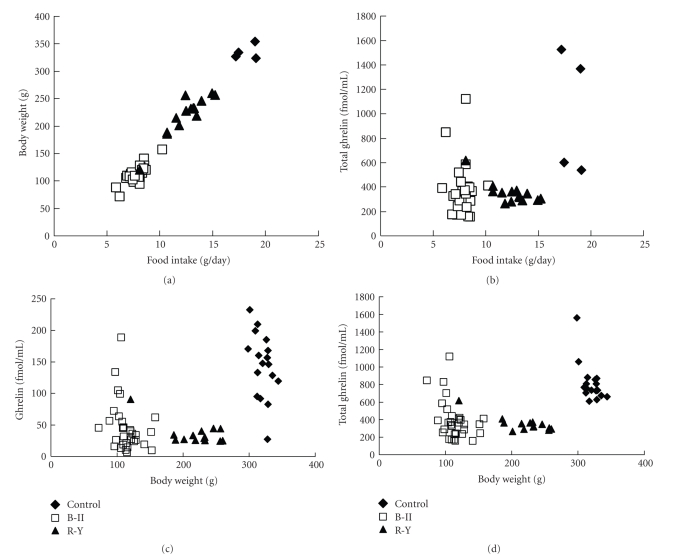
Relationship between daily food intake and body weight (a), between plasma total ghrelin concentrations and daily food intake (b), between plasma ghrelin concentrations and body weight (c), and between plasma total ghrelin concentrations and body weight (d) in rats at 12th week after total gastrectomy combined with B-II or R-Y as compared to the control with sham-operation. Ghrelin concentrations were determined with N-RIA in (c), and total ghrelin concentrations with C-RIA in (b) and (d). Total ghrelin corresponds to the sum of ghrelin and desacyl ghrelin.

The correlation between body weight, 24 hours food intake, and plasma ghrelin concentrations at the 12th postoperative week was studied. Food intake and body weight were correlated with each other irrespective of reconstruction methods and were plotted on a single regression line (Figure 2(a)). In contrast, there was no correlation between food intake and plasma concentrations of total ghrelin (Figure 2(b)). We next examined whether body weight correlates with plasma concentrations of ghrelin or total ghrelin, by using N-RIA and C-RIA. Body weight did not correlate with plasma ghrelin and total ghrelin levels (Figures 2(c) and 2(d)). Furthermore, no significant difference between R-Y and B-II groups was observed in averaged ghrelin levels (B-II: 46.3 ± 7.2 fmol/ml (*n* = 40), R-Y: 35.9 ± 4.6 fmol/ml (*n* = 35)) and total ghrelin levels (B-II: 393.0 ± 40.0 fmol/ml (*n* = 40), R-Y: 349.8 ± 23.2 fmol/ml (*n* = 35)), though some rats in B-II groups showed higher levels of ghrelin and total ghrelin. These results indicate that plasma ghrelin levels are significantly reduced in R-Y and B-II groups and that greater food intake in R-Y than in B-II is related to the method of reconstruction but not plasma ghrelin levels.

### 3.2. Plasma Ghrelin Concentrations and Ghrelin Contents in Duodenum, Jejunum and Pancreas at 12th Postoperative Week

**Figure 3:**
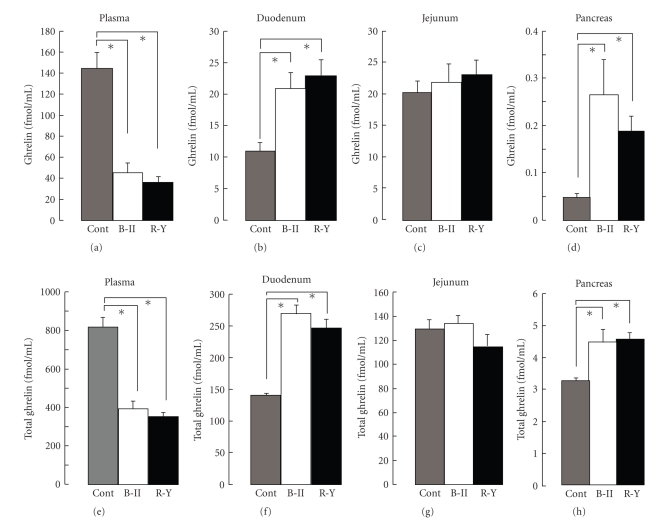
Concentrations of ghrelin and total ghrelin in the plasma, intestine and pancreas after gastrectomy. Ghrelin and total ghrelin concentrations in the plasma ((a), (e)) significantly decreased, while those in the duodenum ((b), (f)) and pancreas ((d), (h)) significantly increased in rats at 12th week after total gastrectomy with B-II or R-Y, compared to the control with sham-operation (cont). In contrast, neither ghrelin (c) nor total ghrelin concentration (g) in the jejunum differed among B-II, R-Y and control groups. Concentrations of ghrelin and total ghrelin were determined by N-RIA and C-RIA, respectively. *N* = 17 for control, 31 for B-II and 14 for R-Y. *P<.05.

Plasma ghrelin concentrations were markedly reduced in both R-Y and B-II groups to the levels of about 30% of those in control rats (Figure 3(a)). Stomach produces approximately 70% of total ghrelin [3]. Therefore, the reduction of plasma ghrelin levels is due to lack of release of ghrelin from stomach. We examined whether the reduction of ghrelin due to gastrectomy could influence the production of ghrelin in the intestine and pancreas, the tissues known to produce ghrelin. Ghrelin concentrations in the duodenum increased approximately by 100% in R-Y and B-II groups (Figure 3(b)), while those in the jejunum did not change (Figure 3(c)). Ghrelin concentrations in the pancreas were much smaller than those in the intestine, however, they dramatically increased approximately by 500% in B-II and by 400% in R-Y groups (Figure 3(d)). On the other hand, plasma concentrations of total ghrelin were approximately five times higher than those of ghrelin, and they were markedly reduced by gastrectomy in both groups (Figure 3(e)). Contents of total ghrelin in the duodenum and pancreas, but not jejunum, increased in both R-Y and B-II groups (Figures 3(f)–3(h)). The gastrectomy-associated relative changes of total ghrelin in the circulation, duodenum and pancreas were similar to those of ghrelin. These results suggest that gastrectomy-induced reductions in circulating ghrelin levels promote synthesis of ghrelin in the duodenum and pancreas, which may have compensatory roles including the restoration of circulating ghrelin levels. In consistence with this inference, plasma levels of ghrelin and total ghrelin that were markedly reduced at 2 weeks after gastrectomy with B-II reconstruction (ghrelin: 25.0 ± 5.3 fmol/ml (*n* = 4), total ghrelin: 295.8 ± 34.6 fmol/ml (*n* = 4)) were significantly (*P* < .05) elevated at the 12th postgastrectomy week (ghrelin: 46.3 ± 7.1 fmol/ml (*n* = 40), total ghrelin: 393.0 ± 40.0  fmol/ml (*n* = 40)).

### 3.3. Effect of Fasting on Plasma Ghrelin Levels at 12th Postoperative Week

**Figure 4:**
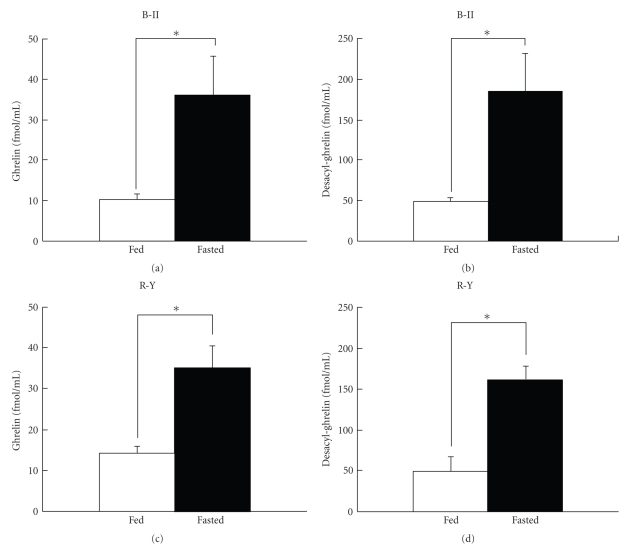
Plasma concentrations of ghrelin and desacyl ghrelin were significantly elevated by fasting. Plasma concentrations of ghrelin ((a), (c)) and desacyl ghrelin ((b), (d)) under fed condition and after 48 hour fasting in B-II group ((a), (b)) and R-Y group ((c), (d)) at 12th postoperative week. Concentrations of ghrelin and desacyl ghrelin were determined by ELISA. *N* = 8 for B-II and 11 for R-Y. *:*P* < .05.

We examined whether reduced levels of plasma ghrelin after gastrectomy could be altered by fasting, the physiological regulator of ghrelin release. At the 12th postoperative week, blood samples were taken from ad-lib fed and 48 hour fasting rats. Effects of fasting on plasma levels of ghrelin and desacyl ghrelin were examined by using ELISA, since RIA did not allow us to determine exact levels of desacyl ghrelin. Plasma ghrelin concentrations were significantly (*P* < .05) elevated by fasting approximately by 3 times in both B-II and R-Y groups (Figures 4(a) and 4(c)). Desacyl ghrelin concentrations were also significantly (*P* < .05) elevated by 3-4 times by fasting in B-II and R-Y groups (Figures 4(b) and 4(d)). Thus, fasting induced similar fold increases in both ghrelin and desacyl ghrelin levels in the circulation in gastrectomized rats.

### 3.4. Effect of Ghrelin Injection on Food Intake in Control and Gastrectomized Rats

**Figure 5:**
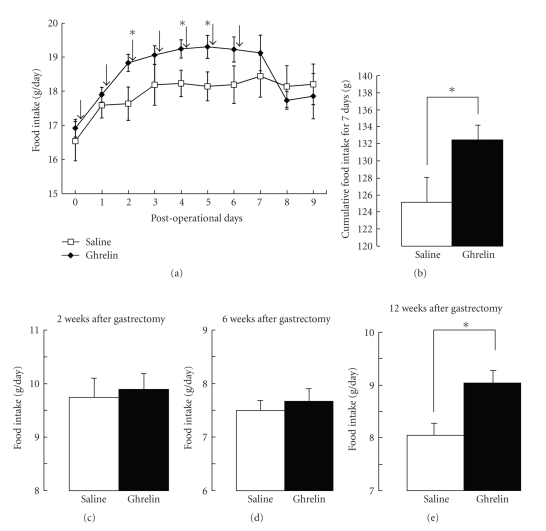
Effect of ghrelin injection on food intake in gastrectomized and control rats. (a) and (b): Ghrelin or saline was injected subcutaneously to control rats once daily for 7 days, and daily food intake was measured for 10 days (a) and expressed by the cumulative food intake for 7 days (b). Arrows indicate the time of injection of ghrelin or saline. *N* = 11 for saline and ghrelin.in (c), (d) and (e): Ghrelin or saline was injected to rats once daily for 7 days at 2 (c), 6 (d) and 12 weeks (e) after total gastrectomy with B-II, and the daily food intake averaged for 7 days is shown. *N* = 21 for saline and 22 for ghrelin in (c). *N* = 21 for saline and 20 for ghrelin in (d). *N* = 37 for saline and ghrelin in (e). *P<0.05.

 Rats aged 8-weeks were divided into two groups and received subcutaneous injection of either saline or ghrelin once a day for seven continuous days. Injection of ghrelin increased food intake, in which significant (*P* < .05) difference was obtained at the 2nd, 4th and 5th days (Figure 5(a)). The cumulative food intake during the treatment period of 7 days was significantly (*P* < .05) increased by ghrelin (Figure 5(b)). 

At 2 weeks after total gastrectomy, rats were divided into two groups and received subcutaneous injection of either saline or ghrelin once a day for 7 continuous days. The 24 hours food intake averaged for the treatment period of 7 days was not significantly different between ghrelin-and saline-treated groups (Figure 5(c)). Essentially the same results were obtained in rats at 6 weeks after total gastrectomy; 24 hours food intake averaged for the treatment period of 7 days was not different between ghrelin-and saline-treated groups (Figure 5(d)).

At the 12th week after total gastrectomy, rats were divided into two groups. One group received daily subcutaneous injection of saline for a week followed by that of ghrelin for the next week. Vice versa, the other group received ghrelin for the first week followed by saline for the next week. The amount of 24 hours food intake in rats injected with ghrelin was significantly (*P* < .05) greater than that with saline (Figure 5(e)). These results indicate that ghrelin injection increases food intake in the later stage of 12 weeks after gastrectomy but not earlier.

## 4. Discussion

 Ghrelin is secreted primarily from the stomach [9, 30], and stimulates food intake and body weight gain. Gastrectomy commonly decreases food intake, body weight, fat mass and bone mass, and is also accompanied by a marked reduction in circulating ghrelin levels [31]. However, whether ghrelin levels are causally related to food intake and/or body weight after gastrectomy is not well understood. In the present study, we produced total gastrectomized rat models and examined postoperative changes in ghrelin levels, food intake and body weight. Plasma ghrelin levels were markedly reduced by gastrectomy irrespective to whether the reconstruction was performed by B-II or R-Y method. However, the recovery from gastrectomy-induced reductions in food intake and body weight was much greater in R-Y than in B-II group. The results indicate that the recovery from reduced food intake and body weight after gastrectomy is not correlated with circulating ghrelin levels but strongly depends on the reconstruction method. 

The mechanism for the better recovery of food intake and body weight with R-Y method after total gastrectomy remains unclear. Ghrelin contents in the duodenum, jejunum and pancreas at 12th postoperative week were not different between B-II and R-Y methods. It is speculated that in B-II method bile juice may directly reflux the esophagojejunostomy and consequently decrease food intake. Alternatively, shorter distance between esophagojejunostomy and jejunojejunostomy in R-Y method than in B-II method may yield smaller reflux of bile juice. It is suggested that selecting the reconstruction method with less reflux of bile juice may contribute to better recovery from the gastrectomy-induced anorexia. 

At the 12th week after gastrectomy, ghrelin production was markedly enhanced in the duodenum, the organ known to produce the second largest amount of ghrelin [3]. This result was essentially the same between B-II and R-Y groups, suggesting that the enhanced ghrelin production is not related to the reconstruction method but likely due to the removal of gastric ghrelin. It is therefore suggested that the enhanced ghrelin production in the duodenum could partly compensate for gastrectomy-associated reductions of the circulating ghrelin and its endocrine functions. In another line of experiments, at 12th postoperative week fasting markedly increased plasma ghrelin levels in gastrectomized rats, indicating that the tissue other than stomach, most likely the duodenum, secretes ghrelin in response to fasting, the physiological regulator of ghrelin secretion [9, 32]. Furthermore, the feeding response to peripheral ghrelin administration was once eliminated by gastrectomy but restored at 12th postoperative week. These data suggest that ghrelin can be released and stimulate food intake under fasted conditions at this later period after gastrectomy. Collectively, the markedly upregulated production of ghrelin in the duodenum could contribute to the recovery of food intake and body weight in the later postoperative period. 

In this study, ghrelin production was also significantly elevated in the pancreas. Since the ghrelin content in the pancreas and its increment due to gastrectomy are much smaller than those in the duodenum, they may neither significantly contribute to the circulating ghrelin levels nor operate endocrine functions. However, previous studies using ghrelin receptor antagonists and ghrelin-deficient mice have demonstrated that ghrelin in the pancreatic islets inhibits insulin release in an autocrine/paracrine manner and consequently regulate blood glucose levels [33, 34]. The late postprandial dumping syndrome accompanying gastrectomy is characterized by hypoglycemia principally due to excessive insulin release. The mechanism for excessive insulin release remains unclear, lack of the stomach-derived ghrelin could be implicated. If so, it is possible that upregulated pancreatic ghrelin compensates lack of stomach-derived ghrelin and attenuate insulin release, thereby counteracting the hypoglycemia in the late dumping syndrome. Further study is necessary to elucidate the role and mechanism for the upregulation of ghrelin production in the pancreas after gastrectomy. 

It has been well documented that administration of pharmacological doses of ghrelin to intact animals increases food intake, induces weight gain, and causes obesity [9, 13–18]. Ghrelin injection failed to stimulate food intake in the early stages of 2nd to 6th week after operation, but increased it in the late stage of 12th week. The results suggest that some machinery that links ghrelin reception to feeding dysfunctions after gastrectomy but can be restored. A candidate for such machinery is the vagal nerve. It has been reported that the peripheral injection of ghrelin does not increase food intake after cutting vagus nerve [15, 30, 35]. It is thought that peripheral ghrelin signal is transmitted to the feeding center at least partly via afferent vagal nerves [15, 30, 35]. In our study, vagus nerves that were cut by the operation of gastrectomy may be regenerated later. It is speculated that regeneration of vagus nerves takes place not immediately but time-dependently, allowing reception of injected ghrelin and transmission of its signal to the feeding center. In addition, chronic hypoghrelinemia in gastrectomized status was reported to induce hypersensitivity to ghrelin as judged by secretion of growth hormone [36]. If this is also the case for the orexigenic action of ghrelin, the hypersensitivity to ghrelin could make the ghrelin injection more potent and/or efficacious in correcting anorexia after gastrectomy. Collectively, in the certain period when vagus nerves are regenerated and sensitivity to ghrelin is elevated, ghrelin treatment is expected to effectively stimulate feeding. In fact, the fractional increase of food intake in response to ghrelin injection at 12th postoperative week was similar to that in control rats (Figures 5(e) versus 5(a)). Thus, our results raise a possibility that ghrelin replacement therapy given in later stages could be effective in promoting food intake and correcting some symptoms associated with gastrectomy in humans.

Another health problem with dysregulated body weight and feeding is obesity. Obesity often causes hyperglycemia, hypertension and/or dyslipidemia, forming metabolic syndrome. Obesity and metabolic syndrome are major risk factors for cardiovascular disease. The rapidly increasing incidence of obesity has become a serious worldwide health problem [37]. An effective treatment of severe obesity is bariatric surgery, in which gastric bypass is a representative method. Gastric bypass, as well as gastrectomy, markedly reduces appetite and body weight, suggesting that changes in plasma ghrelin levels are involved. However, studies on changes in plasma ghrelin levels after gastric bypass have yielded inconsistent results; decreased, unchanged, or increased [38–40]. In our study, better restoration in feeding and body weight after gastrectomy was obtained with R-Y than B-II method, while plasma ghrelin levels were similar. These observations by us and others collectively suggest that feeding and body weight in the earlier period after gastric surgery, are not associated with plasma ghrelin levels but determined by other factors, possibly reconstruction methods associated with specific histological conditions.

In summary, recovery from gastrectomy-induced reductions in food intake and body weight is not related to plasma ghrelin levels but depends on the reconstruction method in rats. At postoperational 12th week, ghrelin production is increased in the duodenum and pancreas, and circulating ghrelin level is elevated by fasting, suggesting that ghrelin could be released from extra-stomach tissues and play a compensatory role. Ghrelin administration stimulates feeding at postoperational 12th week but not earlier, suggesting that ghrelin treatment in later periods could be effective in treating patients with gastrectomy-induced anorexia and associated symptoms.
